# Initial Results of Intra-Annular Self-Expandable Valves: Insights From the OCEAN-TAVI Registry

**DOI:** 10.1016/j.jacasi.2024.04.010

**Published:** 2024-06-11

**Authors:** Shinichi Shirai, Masanori Yamamoto, Fumiaki Yashima, Hirofumi Hioki, Toshinobu Ryuzaki, Toru Morofuji, Toru Naganuma, Futoshi Yamanaka, Kazuki Mizutani, Masahiko Noguchi, Hiroshi Ueno, Kensuke Takagi, Yohei Ohno, Masaki Izumo, Hidetaka Nishina, Hiroto Suzuyama, Kazumasa Yamasaki, Daisuke Hachinohe, Yasushi Fuku, Toshiaki Otsuka, Masahiko Asami, Yusuke Watanabe, Kentaro Hayashida, OCEAN-TAVI Investigators

**Affiliations:** a Department of Cardiology, Kokura Memorial Hospital, Kokura, Japan; b Department of Cardiology, Toyohashi Heart Center, Toyohashi, Japan; c Department of Cardiology, Nagoya Heart Center, Nagoya, Japan; d Department of Cardiology, Gifu Heart Center, Gifu, Japan; e Department of Cardiology, Saiseikai Utsunomiya Hospital, Tochigi, Japan; f Department of Cardiology, IMS Tokyo Katsushika General Hospital, Tokyo, Japan; g Department of Cardiology, Keio University School of Medicine, Tokyo, Japan; h Department of Cardiology, New Tokyo Hospital, Chiba, Japan; i Department of Cardiology, Shonan Kamakura General Hospital, Kanagawa, Japan; j Division of Cardiology, Department of Medicine, Kindai University, Osaka, Japan; k Department of Cardiology, Tokyo Bay Urayasu Ichikawa Medical Center, Urayasu, Japan; l Second Department of Internal Medicine, Toyama University Hospital, Toyama, Japan; m Department of Cardiology, National Cerebral and Cardiovascular Center, Oasaka, Japan; n Department of Cardiology, Tokai University School of Medicine, Isehara, Japan; o Department of Cardiology, St. Marianna University School of Medicine, Tokyo, Japan; p Department of Cardiology, Tsukuba Medical Center Hospital, Tsukuba, Japan; q Division of Cardiology, Saiseikai Kumamoto Hospital Cardiovascular Center, Kumamoto, Japan; r Department of Cardiology, Sapporo Higashi Tokushukai Hospital, Sapporo, Japan; s Cardiovascular Medicine, Sapporo Heart Center, Sapporo Cardio Vascular Clinic, Sapporo, Japan; t Department of Cardiovascular Medicine, Kurashiki Central Hospital, Kurashiki, Japan; u Department of Hygiene and Public Health, Nippon Medical School, Tokyo, Japan; v Center for Clinical Research, Nippon Medical School Hospital, Tokyo, Japan; w Division of Cardiology, Mitsui Memorial Hospital, Tokyo, Japan; x Department of Cardiology, Teikyo University School of Medicine, Tokyo, Japan

**Keywords:** intra-annular, learning curve, PPM, self-expandable valve, TAVR

## Abstract

**Background:**

Navitor, an intra-annular self-expanding heart valve (IA-SEV), is the third transcatheter heart valve introduced in Japan (in April 2022) as the next generation of the Portico valve ahead of other Asian countries.

**Objectives:**

The purpose of this study was to assess the patient–prosthesis mismatch (PPM) after IA-SEV implantation in Asian patients.

**Methods:**

All clinical data were collected from the database of an ongoing prospective Japanese multicenter registry (OCEAN-TAVI [Optimised Catheter Valvular Intervention transcatheter aortic valve implantation]). The primary endpoint was the rate of no PPM; the secondary endpoint included the rate of in-hospital mortality and hemodynamics after IA-SEV implantation.

**Results:**

A total of 463 patients (median age 86; 69.7% female) were enrolled in the registry. The percentages of implanted valves sized 23 mm, 25 mm, 27 mm, and 29 mm were 26.1% (n = 121), 41.7% (n = 193), 22.9% (n = 106), and 9.3% (n = 43), respectively. The primary endpoint of no PPM was achieved in 91.7% of the entire cohort and in 87.3%, 94.2%, 91.4%, and 93.0% of each valve size. The rate of in-hospital mortality was 1.9%. Postprocedural mean pressure gradient was 8.3 ± 4.3 mm Hg. The overall rate of pacemaker implantation was 9.7%; the incidence of pacemaker rate tended to be reduced when dividing the first and second half of operator experiences (13.0% vs. 8.0%; *P* = 0.08).

**Conclusions:**

The initial results for the IA-SEV were excellent regarding hemodynamics and reduction of paravalvular leakage regardless of valve size. The IA-SEV is a useful transcatheter heart valve, especially for Asian patients with a high prevalence of small annulus.

Transcatheter aortic valve replacement (TAVR) was considered to be a beneficial treatment for patients with severe aortic stenosis (AS), and several published studies provided evidence of its effectiveness for any surgical risk patients.^1^, ^2^, ^3^, ^4^, ^5^, ^6^ It is now positioned as a useful treatment for AS comparable to surgical aortic valve replacement. Navitor (Abbott) is an intra-annular self expandable valve (IA-SEV), which was launched in April 2022 as the next-generation device of the Portico valve (Abbott) and the third valve after the SAPIEN (Edwards Lifesciences) and Evolut (Medtronic) series introduced in Japan. The features of IA-SEV include a cylinder-shaped frame, an intra-cellular valve with good hemodynamics, a large cell design useful for coronary artery access, and a NaviSeal skirt (Abbott) that significantly reduces paravalvular leakage (PVL). The delivery system, the FlexNav system (Abbott), is steerable, low profile, and has excellent passability, enabling valve recapture and repositioning.

IA-SEV was first introduced in Japan in Asia and has been successively introduced in other Asian countries. Because Asian individuals are smaller in size and have smaller aortic complexes than Western individuals, IA-SEV was considered to be useful because of its favorable hemodynamic characteristics. We report the early results of patients prospectively enrolled and receiving an IA-SEV implant in a Japanese multicenter large-scale registry.

## Methods

### Study Population

All clinical data were collected from the database of an ongoing prospective Japanese multicenter registry (OCEAN-TAVI [Optimised Catheter Valvular Intervention transcatheter aortic valve implantation]).^7^^,^^8^ This trial is registered with the University Hospital Medical Information Network (UMIN000020423). The study protocol of the OCEAN-TAVI registry was approved by the local institutional review boards of the participating centers, and written informed consent was obtained from all patients before undergoing TAVR. The diagnosis of AS, indications for TAVR, and transcatheter heart valve selection were determined by individual local heart teams. The IA-SEV was launched in April 2022, and 463 patients with AS were treated by TAVR using IA-SEV. In Japan, the indication for this valve is limited to the native valve only; therefore, in this cohort, patients receiving a transcatheter aortic valve in the surgical aortic valve procedure and a transcatheter aortic valve in the transcatheter valve procedure were not enrolled.

### Data Definition and Clinical Endpoints

The OCEAN-TAVI registry data set includes: baseline patient characteristics; laboratory data; echocardiographic data; procedural variables; and clinical outcomes with respect to mortality, rehospitalization, and other clinical adverse events. Information regarding the occurrence and/or causes of adverse events was obtained from either the medical records of each center or treating hospital, or by contacting the patient’s family members. An electronic data capture system was used for the collection of the required materials, and all data were assessed via a self-audit by the site. All clinical endpoints, procedural data, complications, postprocedural parameters, and in-hospital events were defined by using the Valve Academic Research Consortium-3 criteria.^9^

The degree of PVL was classified into 3 grades: nontrivial, mild, and equal or greater than moderate. Postprocedural valve performance was evaluated by using the effective orifice area (EOA), indexed EOA, and incidence of patient–prosthesis mismatch (PPM). The severity of PPM was categorized as no PPM, moderate PPM, or severe PPM according to indexed EOA values and patient obesity. PPM was classified based on the indexed EOA as severe (≤0.65 cm^2^/m^2^) or moderate (0.66 to 0.85 cm^2^/m^2^) in the general population, and as severe (≤0.55 cm^2^/m^2^) or moderate (0.56 to 0.70 cm^2^/m^2^) in the obese population (body mass index ≥30 kg/m^2^).^9^^,^^10^ PPM and postoperative hemodynamics were analyzed based on echocardiographic examinations performed within 1 week of the day after TAVI in all cases.

In the current IA-SEV proctoring system in Japan, operators must perform 3 procedures first with proctor present, and the fourth and later patients can be treated independently. In this analysis, the early experience was defined as the first half of all cases and the late experience as the last half of all cases in the number of cases at each institution. If the number of cases was odd, one more case of early experience was added. All facilities in the proctoring phase with <3 cases were considered to have early experience.

The primary endpoint was the rate of no PPM, and the secondary endpoint was the rate of in-hospital mortality, hemodynamics after implantation, and differences in pacemaker implantation rates between early and late experiences.

### Statistical Analysis

Continuous variables are expressed as mean ± SD and as median (IQR). The Shapiro-Wilk test was used to check for normality of continuous variables. Comparisons between 2 non-normally distributed groups were tested by using the Wilcoxon signed-rank test. The McNemar test was used for comparisons between pretreatment and posttreatment PVL.

All statistical analyses were performed by using JMP version 17.2.0 (SAS Institute, Inc). All statistical tests were 2-sided, and a *P* value <0.05 was considered statistically significant.

## Results

### Baseline Patient Characteristics and Preprocedural Imaging Data

IA-SEV was launched on April 1, 2022; it was implanted in 463 patients enrolled in the OCEAN-TAVI registry through May 31, 2023. The median patient age was 86 (IQR 82-89) years; 69.7% were female (Table 1). Overall, 28.7% of patients were NYHA functional class III or higher, indicating that the devices were implanted in relatively stable patients. The rate of previous pacemaker implantation was 6.4%. The median Society of Thoracic Surgeons score was 5.2 (IQR: 3.6-7.8), and the median B-type natriuretic peptide level was 200.7 (IQR: 91.6-416.3) pg/mL. Preoperative aortic valve area was 0.64 (IQR: 0.51-0.76) cm^2^, index aortic valve area was 0.44 (IQR: 0.36-0.53) cm^2^/m^2^, and mean pressure gradient (mPG) was 42.8 (IQR: 35.2-54.9) mm Hg (Table 2). Implantation was performed in 7 patients with a bicuspid aortic valve. Preoperative aortic regurgitation ≥3° was 6.7% and mitral regurgitation ≥3° was 11.4%. Computed tomography data showed an annulus perimeter of 71.4 ± 6.2 mm and a perimeter-derived diameter of 22.7 ± 2.0 mm. Calcification of the left ventricular outflow tract was mild in 10.7%, moderate in 4.6%, and severe in 2.0%.^11^

**Table 1:** Patient Characteristics (N = 463)

Female	323 (69.7)
Age, y	86 (82-89)
BSA, m^2^	1.43 (1.32-1.55)
NYHA functional class III or higher	133 (28.7)
Hyperlipidemia	259 (55.9)
Diabetes mellitus	136 (29.3)
Hypertension	367 (79.2)
Atrial fibrillation	85 (18.4)
Previous pacemaker	30 (6.4)
Previous myocardial infarction	26 (5.6)
Previous coronary intervention	92 (19.9)
Previous CABG	13 (2.9)
Coronary artery disease	136 (29.4)
Peripheral artery disease	66 (14.3)
Previous stroke	53 (11.0)
Previous ischemic stroke	41 (8.9)
Obstructive pulmonary disease	35 (7.6)
Emergency procedure	18 (3.8)
STS score	5.2 (3.6-7.8)
Chronic kidney disease	317 (68.5)
BNP, pg/mL	200.7 (91.6-416.3)
CRBBB	32 (6.9)
CLBBB	20 (4.8)
In-hospital mortality	9 (1.9)

Values are n (%) or median (IQR).

BNP = B-type natriuretic peptide; BSA = body surface area; CABG = coronary artery bypass grafting; CRBBB = complete right bundle branch block; CLBBB = complete left bundle branch block; STS = Society of Thoracic Surgeons.

**Table 2:** Preprocedural TTE and CT Data

TTE data	
AVA, mm^2^	0.64 (0.51-0.76)
Index AVA, cm^2^/m^2^	0.44 (0.36-0.53)
Mean pressure gradient, mm Hg	42.8 (35.2-54.9)
Maximum velocity, m/s	4.3 (4.0-4.8)
Bicuspid aortic valve	7 (1.5)
LVDd, mm	41.6 (38.0-45.4)
LVDs, mm	26.5 (24.0-31.0)
LVEF (modified Simpson)	63.7 (56.4-67.9)
Preprocedural AR degree ≥3	31 (6.7)
Preprocedural MR degree ≥3	53 (11.4)
Preprocedural TR degree ≥3	40 (8.6)
Moderate to severe pulmonary hypertension	108 (23.4)
CT data	
Annulus area, mm^2^	381.0 (339.2-431.0)
Area-derived annulus diameter, mm	22.0 (20.8-23.4)
Perimeter, mm	70.9 (66.8-75.5)
Perimeter-derived annulus diameter, mm	22.6 (21.3-24.0)
LVOT calcification	
Mild	49 (10.7)
Moderate	21 (4.6)
Severe	9 (2.0)

Values are median (IQR) or n (%).

AR = aortic regurgitation; AVA = aortic valve area; CT = computed tomography; LVDd = Left ventricular diastolic diameter; LVD = left ventricular systolic diameter; LVEF = left ventricular ejection fraction; LVOT = left ventricular outflow tract; MR = mitral regurgitation; TR = tricuspid regurgitation; TTE = transthoracic echocardiogram.

### Procedure and Complication Data

The size of the IA-SEV was 23 mm in 26.1%, 25 mm in 41.7%, 27 mm in 22.9%, and 29 mm in 9.3%. Among all sizes, the numbers of small sized valves (23 mm and 25 mm) totaled 314 cases, accounting for 67.8%. Most of the patients (86.8%) were treated while they were under conscious sedation. A transfemoral approach was used in 97.6% of cases, and 11 (2.4%) patients underwent an alternative approach (trans-subclavian). Coronary artery protection was performed in 1.5% of patients. Overall, 96.8% of patients underwent pre-dilatation, while 3.9% underwent postdilatation. Tamponade was seen in 1 patient, and a second valve was needed in 7 patients because of unfavorable valve position. Among 7 cases requiring a second valve, 2 cases received an implant with other valves (from IA-SEV to balloon-expandable valves). The incidence of ischemic stroke was 2.6%, disabling stroke was 1.3%, all bleeding was 8.9%, acute kidney injury was 4.5% (acute kidney injury stage 3 was 0.4%), and major vascular complications were low at 0.2% (Table 3). New-onset complete left bundle branch block was seen in 24.6%. The rate of pacemaker implantation was 9.7%. The incidence of in-hospital mortality was 1.9%.

**Table 3:** Postprocedural Data and Complications (N = 463)

Valve	
Valve size (intention-to-treat)	
23 mm	121 (26.1)
25 mm	193 (41.7)
27 mm	106 (22.9)
29 mm	43 (9.3)
Conscious sedation	402 (86.8)
Access	
Transfemoral	452 (97.6)
Trans-subclavian	11 (2.4)
Coronary protection	7 (1.5)
Predilatation	448 (96.8)
Postdilatation	18 (3.9)
Emergency PCPS	4 (0.9)
Procedure complication	35 (7.6)
Tamponade	1 (0.2)
Valve embolization	3 (0.6)
Second valve	7 (1.5)
Coronary obstruction	2 (0.4)
Ischemic stroke	12 (2.6)
Disabling stroke	6 (1.3)
All bleeding	41 (8.9)
Life threatening + major bleeding	22 (4.8)
All AKI	21 (4.5)
AKI stage 3	2 (0.4)
All vascular complications	12 (2.6)
Major vascular complications	6 (0.2)
Annulus rupture	2 (0.4)
Surgical conversion	2 (0.4)
New pacemaker	45 (9.7)
New-onset atrial fibrillation	12 (2.6)
New-onset CLBBB	114 (24.6)
In-hospital mortality	9 (1.9)

Values are n (%).

AKI = acute kidney injury; CLBBB = complete left bundle branch block; PCPS = percutaneous cardiopulmonary support.

### Preprocedural and Postprocedural Hemodynamics

**Figure 1:**
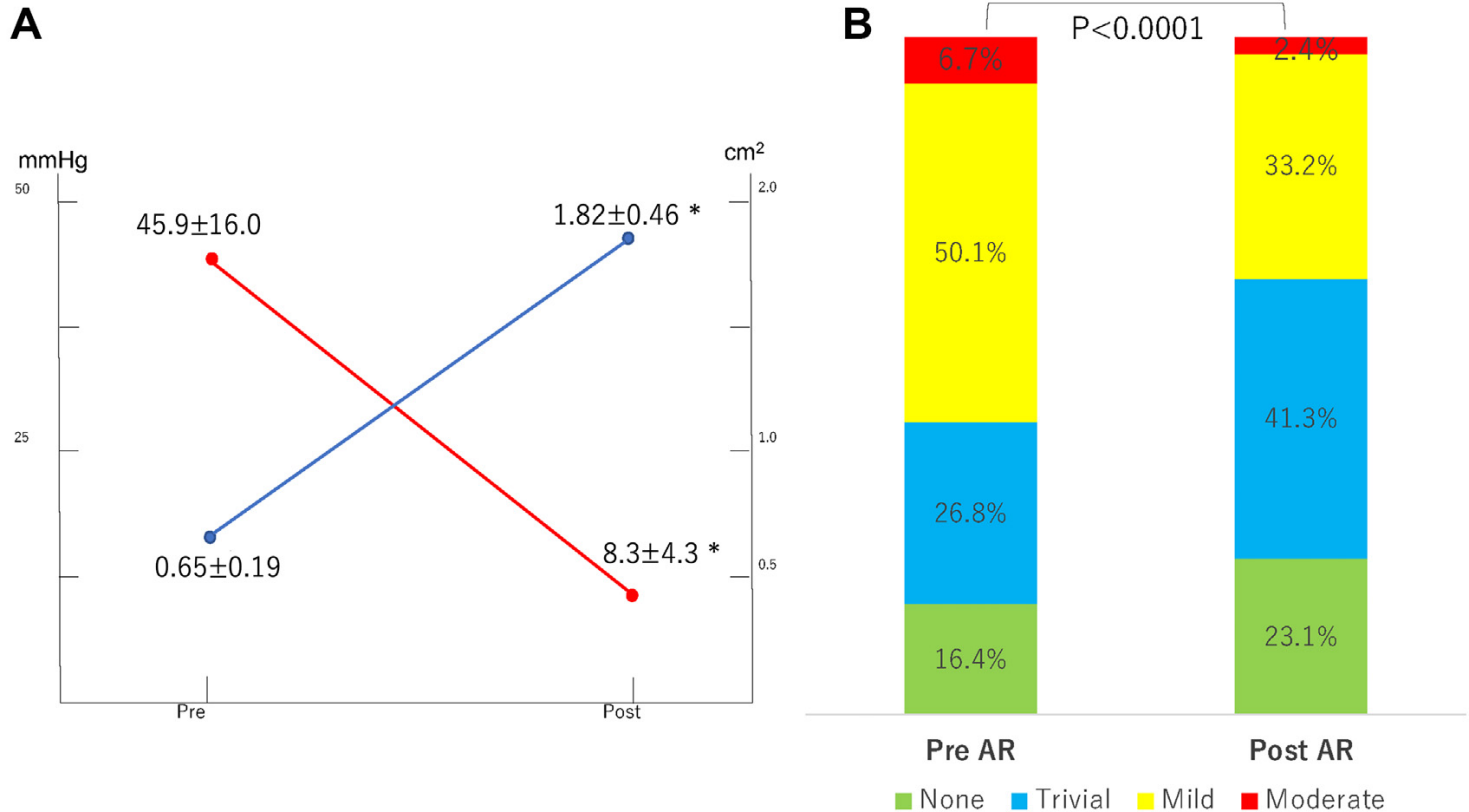
Preprocedural and Postprocedural Hemodynamics and AR and PVL (A) Overall preprocedural and postprocedural hemodynamics: mean pressure gradient and effective orifice area. Compared with baseline, postprocedural hemodynamics significantly improved after the transcatheter aortic valve implantation procedure. (B) Preprocedural and postprocedural paravalvular leakage (PVL). The rate of none/trivial PVL was improved. AR = aortic regurgitation. ∗*P* < 0.0001.

Data are presented on overall hemodynamics and PVL. The EOA and preprocedural and postprocedural mPG findings are shown in Figure 1A. Mean postprocedural EOA was 1.82 ± 0.46 cm^2^ and postprocedural mPG was in single digits (8.3 ± 4.3 mm Hg; compared with preprocedural mPG, *P* < 0.0001). Figure 1B presents the data on preprocedural and postprocedural PVL. After implantation of the valves, the proportion of none and trivial PVL was increased (compared with preprocedural PVL, *P* < 0.0001).

### Preprocedural and Postprocedural Hemodynamics and PPM in Each Size of Valves

EOA increased in proportion to the implanted valve size (Central Illustration) and mPG remained in the single digits for all sizes, indicating good hemodynamics. The proportion of moderate PVL was 0% at 23 mm, 2.1% at 25 mm, 3.8% at 27 mm, and 7.9% at 29 mm. Postoperative hemodynamics (mPG and EOA) for all valve sizes were significantly improved compared with preoperative values, and the percentage of none/trivial aortic regurgitation was also significantly increased compared with preoperative values, clearly indicating a suppressive effect on PVL.

**Central Illustration:**
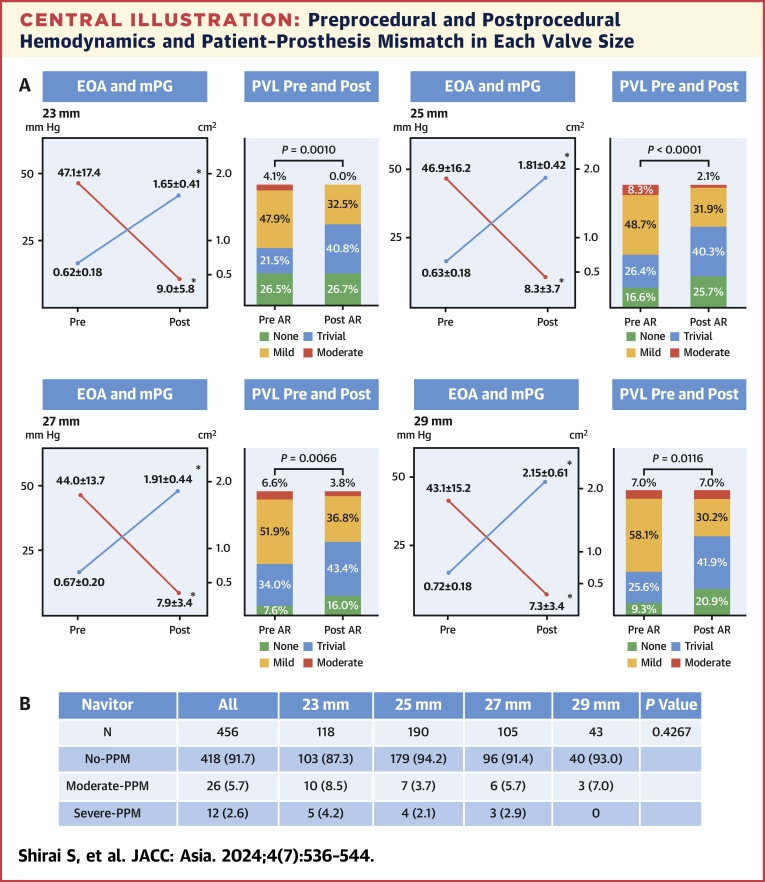
Preprocedural and Postprocedural Hemodynamics and Patient–Prosthesis Mismatch in Each Valve Size (A) Preprocedural and postprocedural hemodynamics and paravalvular leakage (PVL) in each valve size. Each left panel shows the preprocedural/postprocedural mean pressure gradient (mPG) and aortic valve area (effective orifice area [EOA]). Compared with preprocedural findings, hemodynamics (mPG and EOA) and PVL significantly improved after the transcatheter aortic valve implantation procedure in each valve size. ∗*P* < 0.0001. Values are mean ± SD. (B) The percentage of severe patient–prosthesis mismatch (PPM), moderate PPM, and no PPM for each valve. In the entire cohort, severe PPM was 2.6%, and no PPM was 91.7%. For each valve size, the frequency of no PPM was 87.5% for the smallest valve size (23 mm) but reached >90% for larger valves. AR = aortic regurgitation.

PPM after IA-SEV implantation is shown in the Central Illustration. The PPM of the entire cohort was very low, with moderate PPM at 5.7% and severe PPM at 2.6%. The rate of no PPM achieved was 91.7%. The percentage of no PPM increased from 87.5% to 94.2% depending on implanted valve size; moderate PPM was 8.3% and severe PPM was 4.2% even with the smallest valve (23 mm). Even with small size valves, hemodynamics seemed to be excellent.

### 30-Day Pacemaker Learning Curve

**Figure 2:**
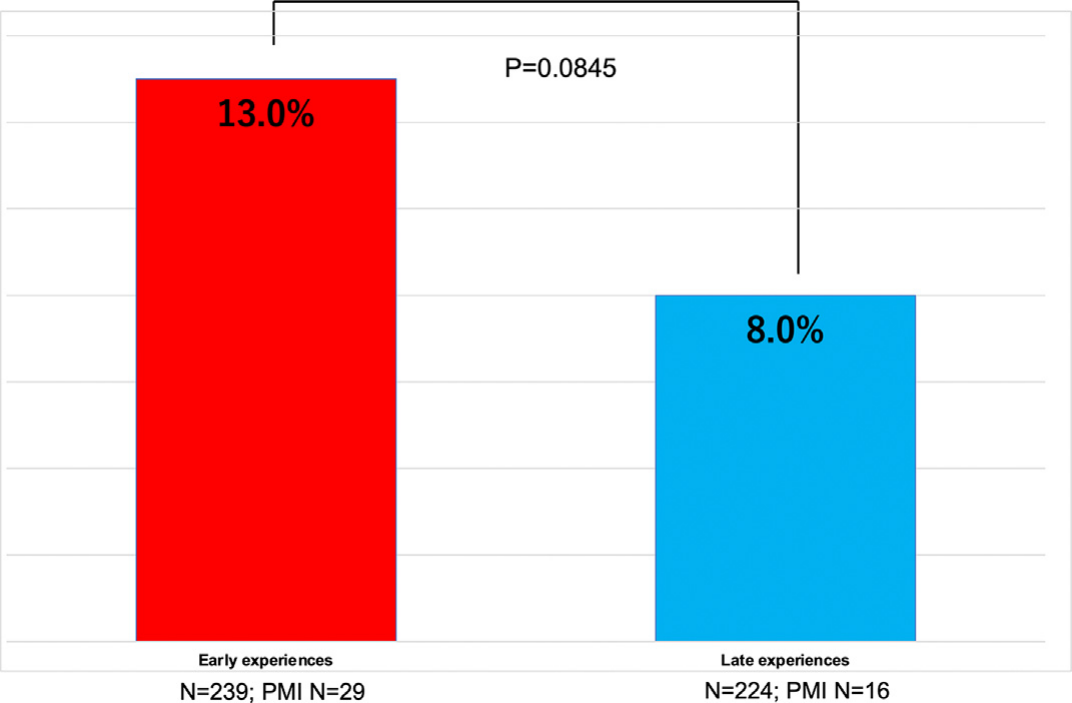
Pacemaker Learning Curve Comparison of the frequency of pacemaker implantation in the early and late experience at each site. The frequency of pacemaker was found to be lower in the late experience compared to the early experience. PMI = pacemaker implantation.

The overall pacemaker rate was 9.7%. However, a comparison of early and late experience at each facility showed that the rate was 13.0% in the early period and 8.0% in the late period (*P* = 0.0845), indicating a learning curve, although it was not statistically significant (Figure 2).

## Discussion

In this report, we present real-world data on IA-SEV from a multicenter registry by an Asian cohort. The main findings are as follows: 1) postprocedural hemodynamics were excellent even in small size valves, and the overall no PPM rate was 91.7% (87.3% in 23 mm valves); 2) the characteristics of the delivery system showed that vascular and hemorrhagic complications were low; 3) the NaviSeal was very effective in reducing PVL, with PVL below mild in >90% of patients in all sizes; and 4) a learning curve was observed for the pacemaker rate, which is expected to decrease as the technique matures.

IA-SEV is the third transcatheter heart valve launched in Japan (after the balloon-expandable valve and the supra-annular self-expandable valve [SA-SEV]). In our results, postprocedural mPG was 9.0 mm Hg at 23 mm, 8.3 mm Hg at 25 mm, 7.9 mm Hg at 27 mm, and 7.3 mm Hg at 29 mm valve sizes. Not only were the values in the single digits for all valve sizes, but they also became smaller as the valve diameter increased. The former generation IA-SEV (PORTICO) was also reported to have good hemodynamics.^12^^,^^13^

The PORTICO IDE study demonstrated the comparison between IA-SEV and commercially available valves including balloon expandable valves and SA-SEV for the patients extreme and high risk of surgery with severe AS.^14^

The results showed that the IA-SEV had better hemodynamics (EOA and mPG) at 2 years compared with the balloon-expandable valve. The SA-SEV and IA-SEV were comparable in hemodynamics after 1 year. The reason why hemodynamics equivalent to SA-SEV can be obtained despite the intra-annular valve type is that the valve platform is cylindrical in shape, which allows the valve to open and close widely.^15^ It is well known that Japanese individuals are clearly smaller than their Western counterparts in terms of body size and valve ring anatomy,^16^ and it has been reported that Japanese patients often receive implants with smaller valves than their Western counterparts.^7^ In our study, almost two thirds of all cases were small size valves (23 mm and 25 mm). Conversely, in a study in Western countries, the proportion of the larger size (27 mm and 29 mm) valves comprised almost 70%.^15^ Our OCEAN-TAVI registry previously reported that the patients with small annulus had remarkably higher residual pressure gradient after implantation of balloon expandable valves.^17^ A recent report also suggests that narrow valve annulus may be a risk for structural valve deterioration in terms of long-term prognosis.^18^ These reports indicate that the use of the smallest IA-SEV (23 mm) provides good hemodynamics and is less likely to produce PPM, characteristics that make it very easy to use for Asian subjects with small body sizes.

The current IA-SEV uses the FlexNav delivery system, which is a hydrophilic-coated, extremely steerable, low-profile system that is easy to deliver. In a clinical trial using this delivery system,^19^ major vascular complications were reportedly 5.0%, which seems to be less than the vascular complications in high-intermediate risk patients in the past.^1^^,^^2^ In this study, all vascular complications were 2.6%, and major vascular complications were 0.2%, which are very low, and we can realize the advantages of this system. The PORTICO NG (Evaluation of the Portico NG [Next Generation] Transcatheter Aortic Valve in High and Extreme Risk Patients With Symptomatic Severe Aortic Stenosis) study^15^ showed that PVL was very well suppressed because of the outer cuff (ie, Naviseal) in IE-SAV. In our study, mild or less PVL was >90% in all sizes, indicating the effectiveness of this treatment. In the PORTICO-I trial,^12^ the grade of PVL was better at 1 year than at 30 days after valve implantation, indicating that PVL decreases with time. Because our data in this study were based on the measurement of PVL immediately after implantation, PVL is expected to decrease further after 30 days and 1 year.

In our report, the rate of pacemaker implantation (PMI) was 9.7%, indicating a learning curve trend. The pacemaker rate for the SA-SEV was 17.4% even in low-risk patients.^6^ In the PORTICO NG trial^15^ in the United States and Europe, the rate was 19.0%. It has been reported that the use of intracardiac echocardiography (ICE) in SA-SEV implantation can significantly reduce the PMI rate.^20^^,^^21^ The participating centers in this study compared centers that implanted valves using ICE (4 centers) with those that did not (19 centers). The PMI rate in the ICE group was 12.4%, while the PMI rate in the non-ICE group was 8.7% (*P* = 0.2257), with no significant difference between the 2 groups. Therefore, for IA-SEV implantation, the use of ICE did not seem to play a role in decreasing the PMI rate. In SA-SEV, a large size valve has been described as an independent determinant of PMI.^22^ We speculate that there are numerous reasons for the lower PMI rate, including the fact that more small sized valves were implanted than recorded in the previous paper,^15^ that many of the institutions participating in the OCEAN-TAVI registry were able to learn implantation techniques from experienced foreign proctors, and that we were able to use the IA-SEV knowledge already obtained in the United States and Europe.

### Study Limitations

Although this study included a relatively large number of patients, the observational and nonrandomized registry data had inherent limitations. First, TAVI valve selection bias was inevitable. Second, the EOA measurement by Doppler echocardiogram could have been affected by technical pitfalls or measurement errors; in addition, accuracy and reproducibility could not be assessed because of a lack of echocardiogram core laboratory.

## Conclusions

The initial results with the IA-SEV were excellent in terms of hemodynamics and reduction of PVL regardless of the valve size. The IA-SEV was a useful transcatheter heart valve, especially for Asian patients with high prevalence of small annulus. There was a learning curve for implantation, and as implantation experience increased, the incidence of pacemaker tended to decrease.

Perspectives **COMPETENCY IN MEDICAL KNOWLEDGE:** The latest-generation IA-SEV has been launched; however, the clinical data and valve hemodynamics for Asian cohorts were not yet clear.

**COMPETENCY IN PATIENT CARE:** The IA-SEV has excellent valve hemodynamics even in smaller size (low incidences of PPM, and low mean pressure gradient). This valve has efficacy for Asian cohorts with small-size anatomy as well as Western cohorts.

**TRANSLATIONAL OUTLOOK:** This study was a short-term observation, but longer term follow-up of the valve will elucidate the hemodynamic benefits on freedom from structural valve deterioration.

## Funding Support and Author Disclosures

The OCEAN-TAVI registry is supported by Edwards Lifesciences, Medtronic, Boston Scientific, Abbott Medical, and Daiichi-Sankyo Company. Drs Takagi and Nishioka are clinical proctors for Edwards Lifesciences. Dr Izumo is a screening proctor for Edwards Lifesciences. Drs Yashima, Ohno, and Asami are clinical proctors for Medtronic. Drs Naganuma, Mizutani, Ueno, and Fuku are clinical proctors for Edwards Lifesciences and Medtronic. Drs Yamamoto, Shirai, Tada, Yamasaki, Hachinohe, Watanabe, and Hayashida are clinical proctors for Edwards Lifesciences, Abbott Medical, and Medtronic. All other authors have reported that they have no relationships relevant to the contents of this paper to disclose.
